# Supplementary material for: Gender-Related Inequality in Childhood Immunization Coverage: A Cross-Sectional Analysis of DTP3 Coverage and Zero-Dose DTP Prevalence in 52 Countries Using the SWPER Global Index
Source: Vaccines (Basel). 2022 Jun 21;10(7):988. doi: 10.3390/vaccines10070988 (PMC9315814; doi:10.3390/vaccines10070988)
Supplement: Supplementary file 1 [file vaccines-10-00988-s001.zip › vaccines-1737788-supplementary figures.pdf]

Figure S1. Zero-dose DTP prevalence by social independence tertile in 52 countries (DHS 2010–2019)

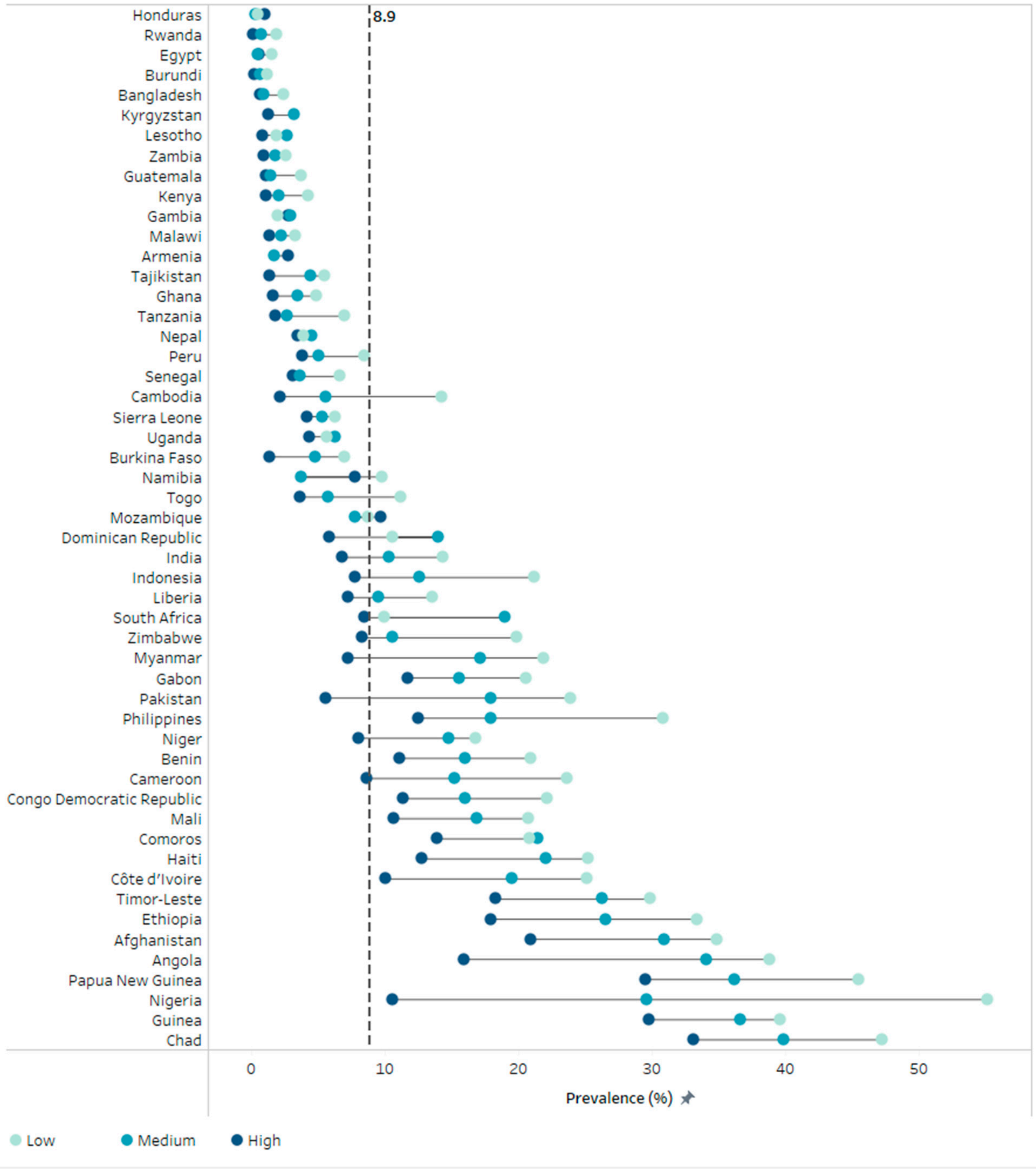

Colored circles indicate social independence tertiles within each country. The black vertical dashed line indicates the median national coverage across 52 countries. DHS=Demographic and Health Surveys. DTP=diphtheria-tetanus-pertussis.

Figure S2. Inequality in zero-dose DTP prevalence in women's social independence in 52 countries: Crude Slope Index of Inequality and Concentration Index (DHS 2010–2019)

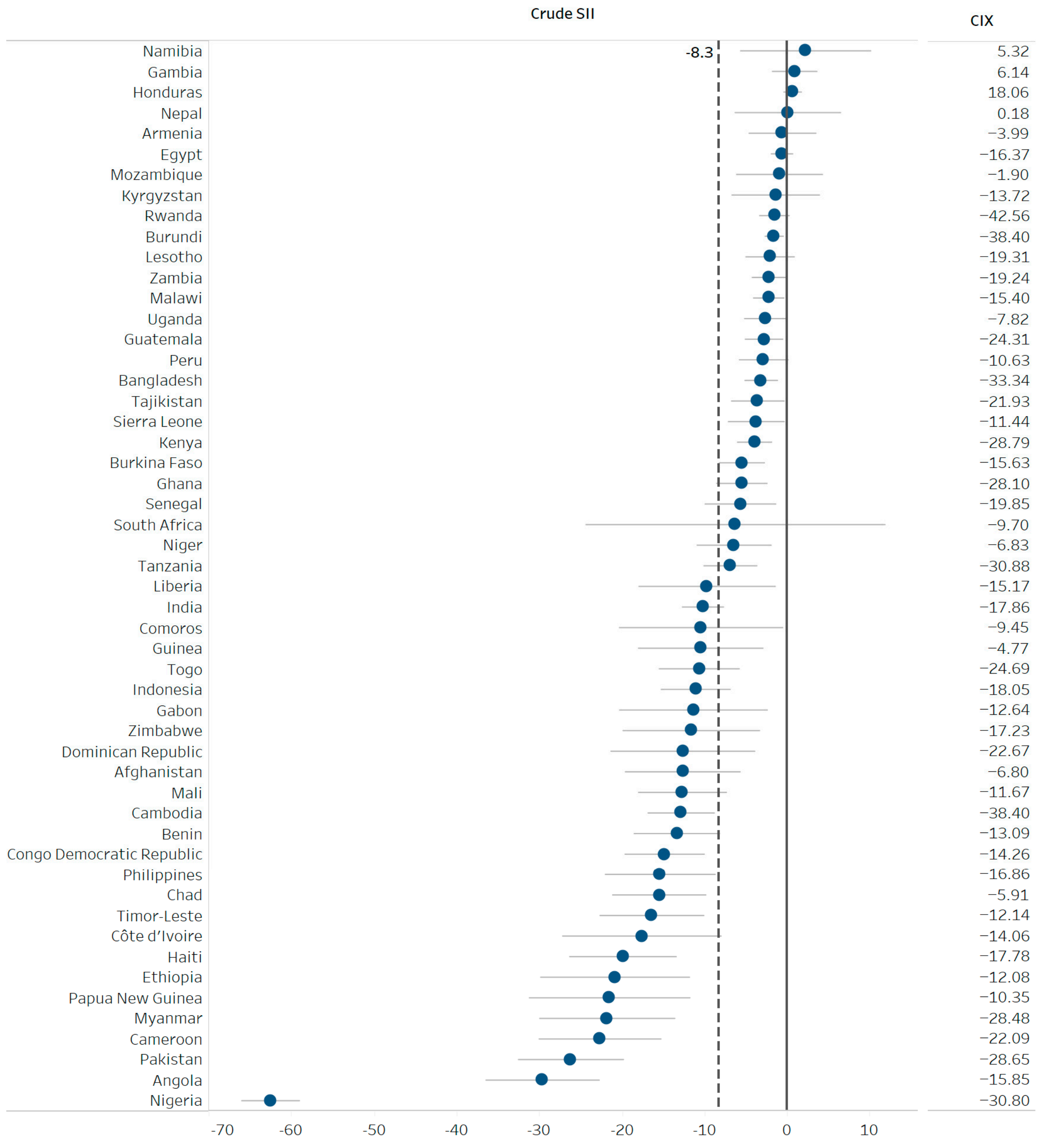

Blue circles show the crude SII for each country. Horizontal grey lines indicate 95% confidence intervals for crude SII estimates. The black vertical dashed line indicates the median crude SII value across 52 countries. The black vertical solid line indicates the SII value of no inequality (zero). CIX=Concentration Index. DHS=Demographic and Health Surveys. DTP=diphtheria-tetanus-pertussis. SII=Slope Index of Inequality.
