# Supplementary material for: Gender-Related Inequality in Childhood Immunization Coverage: A Cross-Sectional Analysis of DTP3 Coverage and Zero-Dose DTP Prevalence in 52 Countries Using the SWPER Global Index
Source: Vaccines (Basel). 2022 Jun 21;10(7):988. doi: 10.3390/vaccines10070988 (PMC9315814; doi:10.3390/vaccines10070988)
Supplement: Supplementary file 1 [file vaccines-10-00988-s001.zip › vaccines-1737788-supplementary tables.pdf]

Table S1. Survey information, SWPER Global Index social independence domain frequencies, and mean social independence score for 52 LMIC countries.

Country	Year	Age (months)	Total N	Low social independence tertile N	Medium social independence tertile N	High social independence tertile N	Mean social independence score	LL	UL
Afghanistan	2015	12 - 35	12088	6334	4456	1298	-0.497	-0.531	-0.463
Angola	2015	12 - 35	3708	1725	1294	689	-0.288	-0.339	-0.237
Armenia	2015	12 - 35	662	7	151	504	0.912	0.840	0.983
Bangladesh	2017	12 - 35	3257	1438	1344	475	-0.396	-0.428	-0.365
Benin	2017	12 - 35	4342	1947	1604	791	-0.379	-0.412	-0.346
Burkina Faso	2010	12 - 35	5215	3278	1513	424	-0.639	-0.662	-0.616
Burundi	2016	12 - 35	4517	1170	2190	1157	-0.112	-0.141	-0.083
Cambodia	2014	12 - 35	2667	395	1097	1175	0.259	0.215	0.303
Cameroon	2018	12 - 35	2778	1166	950	662	-0.291	-0.348	-0.234
Chad	2014	12 - 35	5362	3888	1164	310	-0.836	-0.863	-0.809
Comoros	2012	12 - 35	1024	400	287	337	-0.050	-0.128	0.028
Côte d'Ivoire	2011	12 - 35	2174	1217	685	272	-0.510	-0.565	-0.455
Democratic Republic of the Congo	2013	12 - 35	5784	2522	2289	973	-0.324	-0.362	-0.285
Dominican Republic	2013	18 - 35	795	142	288	365	0.473	0.355	0.591
Egypt	2014	18 - 35	4688	675	1567	2446	0.357	0.319	0.395
Ethiopia	2016	12 - 35	3596	2094	1052	450	-0.585	-0.636	-0.535
Gabon	2012	12 - 35	1481	461	625	395	0.118	0.023	0.214
Gambia	2013	12 - 35	2746	1533	776	437	-0.470	-0.519	-0.422
Ghana	2014	12 - 35	1948	674	678	596	0.061	-0.017	0.139
Guatemala	2014	12 - 35	4192	1140	1599	1453	0.041	0.001	0.081
Guinea	2018	12 - 35	2459	1447	672	340	-0.602	-0.644	-0.560
Haiti	2016	12 - 35	2091	483	793	815	0.238	0.176	0.301
Honduras	2011	12 - 35	3480	1107	1363	1010	0.002	-0.037	0.040
India	2015	12 - 35	16946	3397	6384	7165	0.231	0.210	0.251
Indonesia	2017	12 - 35	6660	568	1966	4126	0.666	0.631	0.702
Kenya	2014	12 - 35	3254	1002	1326	926	0.040	-0.007	0.087
Kyrgyzstan	2012	18 - 35	1169	6	248	915	0.829	0.765	0.894
Lesotho	2014	12 - 35	825	69	421	335	0.304	0.229	0.379
Liberia	2019	12 - 35	1311	634	473	204	-0.314	-0.379	-0.250
Malawi	2015	12 - 35	5306	1968	2392	946	-0.296	-0.324	-0.268
Mali	2018	12 - 35	3398	1985	1008	405	-0.593	-0.628	-0.559

Mozambique	2011	12 - 35	3408	1642	1263	503	-0.447	-0.482	-0.413
Myanmar	2015	12 - 35	1698	255	605	838	0.462	0.388	0.537
Namibia	2013	12 - 35	867	133	236	498	0.650	0.554	0.745
Nepal	2016	12 - 35	1954	702	772	480	-0.172	-0.235	-0.108
Niger	2012	12 - 35	4246	3169	830	247	-0.870	-0.894	-0.845
Nigeria	2018	12 - 35	11046	5271	2665	3110	-0.253	-0.294	-0.212
Pakistan	2017	12 - 35	4663	1312	1300	1210	0.047	-0.016	0.110
Papua New Guinea	2016	12 - 35	3017	605	1049	1363	0.131	0.078	0.185
Peru	2019	18 - 35	5256	491	1569	3196	0.794	0.754	0.835
Philippines	2017	12 - 35	3724	307	1174	2243	0.692	0.639	0.744
Rwanda	2014	12 - 35	2487	293	987	1207	0.313	0.280	0.347
Senegal	2019	12 - 35	2100	975	718	407	-0.224	-0.301	-0.147
Sierra Leone	2019	12 - 35	2945	1394	1017	534	-0.409	-0.443	-0.375
South Africa	2016	12 - 35	512	29	122	361	0.881	0.779	0.983
Tajikistan	2012	18 - 35	1559	67	562	930	0.474	0.428	0.521
Timor-Leste	2016	12 - 35	2692	461	919	1312	0.340	0.294	0.385
Togo	2013	12 - 35	2392	909	959	524	-0.191	-0.246	-0.136
Uganda	2016	12 - 35	4779	1734	2049	996	-0.199	-0.232	-0.165
United Republic of Tanzania	2015	12 - 35	3374	955	1482	937	-0.096	-0.137	-0.055
Zambia	2018	12 - 35	2827	979	1273	575	-0.184	-0.227	-0.142
Zimbabwe	2015	12 - 35	1917	328	835	754	0.134	0.088	0.180

Table S2. Zero-DTP prevalence overall and by SWPER Global Index social independence domain, children age 12-35 months in 52 LMIC countries.

Country	National average			Low social independence tertile			Medium social independence tertile			High social independence tertile		
	Prevalence	LL	UL	Prevalence	LL	UL	Prevalence	LL	UL	Prevalence	LL	UL
Overall median	*8.9%*	*5.1%*	*14.3%*	*12.4%*	*7.0%*	*20.8%*	*8.6%*	*4.6%*	*15.5%*	*6.3%*	*3.2%*	*8.4%*
Afghanistan	31.7%	28.1%	35.5%	34.9%	30.4%	39.6%	30.9%	26.7%	35.5%	21.0%	16.2%	26.6%
Angola	32.1%	29.2%	35.2%	38.9%	34.8%	43.1%	34.1%	30.3%	38.1%	16.0%	12.9%	19.7%
Armenia	2.5%	1.4%	4.3%	--	--	--	1.7%	0.5%	5.8%	2.8%	1.6%	4.8%
Bangladesh	1.6%	1.0%	2.5%	2.5%	1.5%	3.9%	0.9%	0.5%	1.7%	0.7%	0.3%	1.6%
Benin	17.4%	15.5%	19.4%	20.9%	18.2%	24.0%	16.0%	13.8%	18.5%	11.1%	8.9%	13.8%
Burkina Faso	5.9%	4.9%	7.2%	7.0%	5.6%	8.7%	4.8%	3.5%	6.6%	1.4%	0.7%	3.0%
Burundi	0.7%	0.5%	1.0%	1.2%	0.7%	2.1%	0.6%	0.4%	1.1%	0.2%	0.1%	0.7%
Cambodia	5.4%	4.3%	6.8%	14.3%	10.6%	19.0%	5.6%	4.1%	7.6%	2.2%	1.3%	3.7%
Cameroon	17.4%	14.9%	20.2%	23.6%	19.6%	28.2%	15.3%	12.2%	18.9%	8.7%	6.2%	11.9%
Chad	44.7%	42.1%	47.4%	47.2%	44.4%	50.0%	39.9%	36.1%	43.8%	33.1%	26.4%	40.6%
Comoros	18.8%	15.3%	22.9%	20.8%	15.9%	26.8%	21.5%	15.2%	29.5%	13.9%	9.6%	19.8%
Côte d'Ivoire	21.3%	18.2%	24.9%	25.1%	20.7%	30.2%	19.5%	15.5%	24.3%	10.1%	6.7%	15.0%
Democratic Republic of the Congo	17.7%	15.6%	20.0%	22.2%	19.3%	25.3%	16.0%	13.5%	18.8%	11.3%	8.4%	15.1%
Dominican Republic	9.3%	6.9%	12.6%	10.6%	6.0%	17.8%	14.0%	9.5%	20.2%	5.8%	3.3%	10.1%
Egypt	0.7%	0.4%	1.1%	1.5%	0.6%	3.7%	0.5%	0.2%	1.1%	0.6%	0.3%	1.1%
Ethiopia	29.5%	26.5%	32.7%	33.4%	29.9%	37.0%	26.5%	21.6%	32.1%	17.9%	12.0%	26.0%
Gabon	15.3%	11.7%	19.8%	20.6%	14.4%	28.6%	15.6%	10.6%	22.3%	11.7%	7.4%	18.1%
Gambia	2.5%	1.7%	3.5%	2.0%	1.3%	3.1%	3.0%	1.8%	5.1%	2.8%	1.5%	5.3%
Ghana	3.3%	2.3%	4.5%	4.9%	3.3%	7.2%	3.5%	2.1%	5.8%	1.6%	0.9%	2.8%
Guatemala	2.0%	1.5%	2.6%	3.7%	2.5%	5.4%	1.5%	0.9%	2.4%	1.1%	0.5%	2.1%
Guinea	37.4%	34.0%	40.9%	39.6%	35.6%	43.7%	36.7%	31.8%	41.8%	29.8%	24.6%	35.5%
Haiti	19.0%	16.3%	22.0%	25.2%	20.6%	30.6%	22.1%	18.0%	26.9%	12.8%	9.9%	16.4%
Honduras	0.6%	0.4%	1.0%	0.5%	0.2%	1.1%	0.4%	0.1%	1.2%	1.0%	0.5%	2.0%
India	9.6%	8.9%	10.4%	14.4%	12.9%	16.0%	10.4%	9.3%	11.5%	6.8%	5.8%	7.9%
Indonesia	10.4%	9.3%	11.6%	21.2%	16.8%	26.2%	12.6%	10.4%	15.2%	7.7%	6.8%	8.8%
Kenya	2.3%	1.8%	3.0%	4.2%	3.1%	5.9%	2.1%	1.4%	3.3%	1.1%	0.6%	2.1%
Kyrgyzstan	1.8%	1.0%	3.2%	--	--	--	3.2%	1.3%	8.1%	1.3%	0.6%	2.7%
Lesotho	1.8%	1.1%	3.1%	1.9%	0.3%	12.7%	2.6%	1.4%	5.1%	0.8%	0.3%	2.5%
Liberia	10.8%	8.5%	13.7%	13.6%	9.9%	18.3%	9.6%	6.5%	13.9%	7.3%	4.6%	11.3%
Malawi	2.5%	2.0%	3.1%	3.3%	2.4%	4.5%	2.2%	1.6%	3.0%	1.4%	0.7%	2.6%

Mali	18.5%	16.0%	21.3%	20.8%	17.6%	24.4%	16.9%	14.0%	20.3%	10.7%	7.4%	15.2%
Mozambique	8.5%	7.0%	10.3%	8.7%	6.9%	10.9%	7.7%	5.8%	10.2%	9.7%	6.4%	14.5%
Myanmar	12.8%	10.2%	15.8%	21.9%	15.6%	29.9%	17.1%	12.7%	22.7%	7.3%	5.1%	10.2%
Namibia	7.0%	5.2%	9.5%	9.8%	4.8%	18.9%	3.7%	1.6%	8.3%	7.8%	5.3%	11.2%
Nepal	4.0%	2.7%	6.0%	4.0%	2.3%	6.8%	4.5%	2.7%	7.4%	3.5%	1.5%	8.0%
Niger	16.1%	13.9%	18.4%	16.8%	14.6%	19.3%	14.8%	11.8%	18.4%	8.1%	4.4%	14.4%
Nigeria	36.5%	34.7%	38.4%	55.2%	52.8%	57.6%	29.6%	27.4%	31.9%	10.6%	9.4%	12.0%
Pakistan	15.3%	12.7%	18.4%	24.0%	19.6%	29.0%	17.9%	13.6%	23.2%	5.6%	4.0%	7.7%
Papua New Guinea	35.6%	32.3%	39.0%	45.5%	38.7%	52.5%	36.2%	31.8%	40.8%	29.5%	25.4%	34.1%
Peru	4.6%	3.8%	5.4%	8.5%	5.8%	12.4%	5.1%	3.8%	6.7%	3.8%	3.0%	4.9%
Philippines	15.5%	13.7%	17.4%	30.8%	23.9%	38.8%	18.0%	14.7%	21.8%	12.5%	10.8%	14.5%
Rwanda	0.6%	0.2%	1.5%	1.9%	0.7%	5.1%	0.8%	0.3%	2.4%	0.1%	0.0%	0.6%
Senegal	4.8%	3.4%	6.7%	6.7%	4.5%	9.8%	3.7%	2.4%	5.6%	3.1%	1.5%	6.4%
Sierra Leone	5.6%	4.5%	6.9%	6.3%	4.8%	8.2%	5.3%	3.9%	7.2%	4.2%	2.6%	6.9%
South Africa	11.0%	7.6%	15.8%	10.0%	2.2%	35.0%	19.0%	9.5%	34.3%	8.5%	5.1%	13.8%
Tajikistan	2.7%	1.9%	3.9%	5.5%	1.9%	15.2%	4.5%	2.8%	7.1%	1.4%	0.8%	2.6%
Timor-Leste	23.0%	20.5%	25.7%	29.9%	24.9%	35.4%	26.2%	22.6%	30.2%	18.3%	15.7%	21.2%
Togo	7.2%	5.6%	9.2%	11.2%	8.6%	14.3%	5.8%	4.3%	7.8%	3.6%	2.0%	6.5%
Uganda	5.7%	4.8%	6.7%	5.7%	4.5%	7.2%	6.3%	4.7%	8.4%	4.3%	3.1%	6.1%
United Republic of Tanzania	3.7%	2.7%	5.0%	7.0%	4.9%	10.0%	2.7%	1.7%	4.2%	1.8%	1.1%	3.1%
Zambia	1.9%	1.3%	2.8%	2.6%	1.7%	4.1%	1.8%	1.0%	3.3%	0.9%	0.4%	2.4%
Zimbabwe	11.4%	9.2%	14.0%	19.9%	13.8%	27.8%	10.6%	8.1%	13.8%	8.3%	5.9%	11.7%

Table S3. Zero-DTP measures of inequality by SWPER Global Index social independence domain, children age 12-35 months in 52 LMIC countries.

Country	Crude SII	LL	UL	Adjusted SII	LL	UL	CIX	LL	UL
Overall median	*-8.3*	*-11.5*	*-3.9*	*-3.5*	*-5.0*	*-2.2*	*-15.5*	*-18.0*	*-12.3*
Afghanistan	-12.7	-19.6	-5.7	-2.5	-5.4	0.4	-6.8	-10.6	-3.0
Angola	-29.6	-36.5	-22.8	-8.7	-19.3	1.9	-15.8	-19.5	-12.2
Armenia	-0.6	-4.6	3.5	-0.1	-1.3	1.2	-4.0	-31.3	23.3
Bangladesh	-3.2	-5.1	-1.2	-3.4	-6.7	-0.1	-33.3	-45.3	-21.3
Benin	-13.4	-18.5	-8.2	-3.1	-11.4	5.1	-13.1	-17.6	-8.6
Burkina Faso	-5.5	-8.2	-2.8	-1.7	-4.1	0.8	-15.6	-22.3	-8.9
Burundi	-1.6	-2.7	-0.5	-2.2	-5.6	1.1	-38.4	-56.2	-20.6
Cambodia	-12.9	-16.9	-8.9	-5.2	-11.5	1.0	-38.4	-48.9	-27.9
Cameroon	-22.7	-30.1	-15.3	-8.0	-22.0	5.9	-22.1	-28.0	-16.2
Chad	-15.5	-21.2	-9.9	-11.5	-18.5	-4.5	-5.9	-8.2	-3.7
Comoros	-10.5	-20.3	-0.6	-5.1	-19.0	8.8	-9.4	-18.3	-0.6
Côte d'Ivoire	-17.6	-27.2	-8.0	-16.0	-30.1	-1.8	-14.1	-21.1	-7.0
Democratic Republic of the Congo	-14.9	-19.7	-10.1	-2.9	-7.2	1.3	-14.3	-18.7	-9.8
Dominican Republic	-12.7	-21.4	-4.0	-3.9	-7.3	-0.4	-22.7	-38.3	-7.0
Egypt	-0.7	-2.0	0.6	-0.4	-1.2	0.4	-16.4	-46.1	13.4
Ethiopia	-20.9	-29.9	-11.9	-6.8	-16.9	3.2	-12.1	-17.6	-6.6
Gabon	-11.4	-20.3	-2.4	-13.3	-25.8	-0.9	-12.6	-23.1	-2.2
Gambia	0.9	-1.8	3.6	-1.8	-8.9	5.4	6.1	-11.8	24.1
Ghana	-5.5	-8.6	-2.5	-2.3	-5.2	0.6	-28.1	-39.5	-16.7
Guatemala	-2.8	-5.1	-0.6	-3.6	-7.7	0.5	-24.3	-42.3	-6.3
Guinea	-10.5	-18.0	-3.0	-4.5	-14.2	5.3	-4.8	-8.2	-1.3
Haiti	-19.9	-26.4	-13.5	-5.2	-20.5	10.1	-17.8	-23.2	-12.3
Honduras	0.6	-0.4	1.7	1.4	-1.8	4.6	18.1	-9.1	45.2
India	-10.2	-12.7	-7.7	-4.2	-8.9	0.4	-17.9	-22.3	-13.5
Indonesia	-11.1	-15.3	-6.9	-16.4	-26.8	-5.9	-18.0	-23.9	-12.2
Kenya	-4.0	-6.0	-1.9	-1.1	-3.0	0.8	-28.8	-42.6	-15.0
Kyrgyzstan	-1.4	-6.7	3.9	-1.5	-4.0	1.1	-13.7	-63.5	36.1
Lesotho	-2.1	-5.0	0.8	-2.8	-15.5	10.0	-19.3	-42.4	3.8
Liberia	-9.7	-18.0	-1.5	-4.3	-10.3	1.6	-15.2	-27.6	-2.7
Malawi	-2.3	-4.1	-0.4	-2.7	-5.9	0.6	-15.4	-27.2	-3.6
Mali	-12.7	-18.0	-7.4	-5.3	-13.9	3.4	-11.7	-16.3	-7.1
Mozambique	-0.9	-6.1	4.2	-0.8	-3.8	2.1	-1.9	-12.3	8.5

Myanmar	-21.8	-30.0	-13.6	-26.8	-44.5	-9.0	-28.5	-36.7	-20.3
Namibia	2.2	-5.7	10.1	-0.4	-1.3	0.5	5.3	-13.5	24.1
Nepal	0.1	-6.3	6.4	0.0	-0.3	0.3	0.2	-26.6	26.9
Niger	-6.4	-10.9	-2.0	-4.8	-12.9	3.2	-6.8	-11.5	-2.1
Nigeria	-62.6	-66.1	-59.1	-30.5	-37.2	-23.7	-30.8	-32.5	-29.2
Pakistan	-26.2	-32.6	-19.8	-8.4	-21.5	4.8	-28.7	-34.1	-23.2
Papua New Guinea	-21.5	-31.2	-11.8	-12.0	-23.3	-0.7	-10.3	-15.1	-5.6
Peru	-2.9	-5.8	0.1	-1.4	-4.3	1.5	-10.6	-21.6	0.3
Philippines	-15.4	-22.0	-8.7	-12.8	-26.9	1.3	-16.9	-23.4	-10.3
Rwanda	-1.6	-3.4	0.2	-0.7	-1.3	-0.1	-42.6	-64.1	-21.0
Senegal	-5.7	-10.0	-1.4	-0.6	-10.4	9.1	-19.9	-31.8	-7.9
Sierra Leone	-3.8	-7.1	-0.4	-5.2	-13.0	2.5	-11.4	-21.3	-1.6
South Africa	-6.3	-24.4	11.8	4.0	-21.8	29.7	-9.7	-37.3	17.9
Tajikistan	-3.6	-6.8	-0.4	-13.1	-38.1	11.9	-21.9	-38.7	-5.2
Timor-Leste	-16.4	-22.7	-10.1	-5.8	-14.6	3.0	-12.1	-16.7	-7.6
Togo	-10.7	-15.5	-5.8	-1.5	-3.8	0.8	-24.7	-35.5	-13.9
Uganda	-2.6	-5.2	0.0	0.1	-2.9	3.0	-7.8	-15.7	0.0
United Republic of Tanzania	-6.9	-10.1	-3.7	-4.5	-11.3	2.3	-30.9	-41.4	-20.3
Zambia	-2.2	-4.3	-0.1	-0.6	-3.9	2.7	-19.2	-36.4	-2.0
Zimbabwe	-11.6	-19.9	-3.4	-0.2	-4.2	3.8	-17.2	-28.4	-6.1

Table S4. Measures of inequality in DTP3 coverage by SWPER Global Index social independence domain, children age 12-23 months.

Country	Crude SII	LL	UL	Adjusted SII	LL	UL	CIX	LL	UL
Afghanistan	16.0	6.1	25.9	8.0	1.04	14.97	4.7	1.9	7.5
Angola	34.5	23.7	45.2	14.4	-0.35	29.15	14.8	10.2	19.4
Armenia	-3.7	-13.3	5.8	-1.1	-5.78	3.52	-0.7	-2.4	1.1
Bangladesh	4.9	1.1	8.6	5.1	-2.46	12.57	0.9	0.2	1.5
Benin	13.2	5.6	20.7	3.2	-8.44	14.89	3.1	1.3	4.9
Burkina Faso	5.0	-0.1	10.1	0.0	-3.32	3.27	0.9	0.0	1.9
Burundi	1.1	-1.2	3.4	2.5	-5.35	10.43	0.2	-0.2	0.6
Cambodia	18.7	10.4	27.0	9.1	-4.76	22.94	3.7	2.1	5.4
Cameroon	29.4	19.7	39.1	10.2	-5.62	26.09	7.1	4.6	9.6
Chad	5.6	-3.2	14.4	0.2	-4.88	5.35	2.9	-1.8	7.6
Comoros	20.8	6.3	35.2	4.2	-23.25	31.55	4.8	1.4	8.3
Côte d'Ivoire	16.4	2.3	30.6	5.8	-11.19	22.85	4.5	0.6	8.4
Democratic Republic of the Congo	25.3	16.4	34.2	15.5	4.05	26.85	7.1	4.5	9.7
Dominican Republic	19.2	4.0	34.5	20.4	-0.53	41.32	4.2	0.9	7.5
Egypt	5.5	2.1	8.9	6.4	-2.66	15.56	0.9	0.4	1.5
Ethiopia	33.0	20.4	45.7	13.0	-2.75	28.74	10.5	6.3	14.8
Gabon	9.6	-8.7	27.9	-0.3	-16.57	16.02	2.1	-1.9	6.2
Gambia	-4.6	-12.9	3.7	-4.9	-24.11	14.34	-0.9	-2.4	0.7
Ghana	7.1	-0.7	14.9	11.6	-10.66	33.87	1.3	-0.1	2.8
Guatemala	13.3	6.3	20.4	3.8	-6.91	14.43	2.7	1.2	4.1
Guinea	9.4	0.2	18.6	2.3	-6.82	11.50	4.0	0.0	7.9
Haiti	36.3	26.0	46.5	10.7	-3.71	25.11	11.2	7.8	14.7
Honduras	0.3	-3.2	3.7	-6.3	-21.13	8.49	0.0	-0.6	0.7
India	18.1	14.1	22.1	9.7	1.20	18.17	3.8	3.0	4.7
Indonesia	18.6	11.8	25.4	14.9	3.36	26.39	4.1	2.6	5.7
Kenya	10.6	3.6	17.7	2.3	-4.86	9.41	2.0	0.7	3.3
Kyrgyzstan	-5.3	-16.9	6.3	0.1	-0.81	1.09	-1.0	-3.3	1.2
Lesotho	-1.6	-14.4	11.2	-13.3	-48.44	21.76	-0.3	-2.8	2.2
Liberia	-8.9	-25.2	7.4	-22.5	-45.56	0.54	-2.2	-6.3	1.9
Malawi	7.0	2.5	11.5	7.5	0.10	15.00	1.3	0.5	2.1
Mali	12.1	4.0	20.1	4.7	-5.87	15.21	2.9	0.9	4.9
Mozambique	-1.0	-10.5	8.5	-2.4	-13.23	8.43	-0.2	-2.3	1.9
Myanmar	39.9	27.5	52.3	29.4	9.74	49.04	10.9	7.0	14.8

Namibia	-4.7	-21.2	11.8	6.2	-15.87	28.20	-0.9	-4.2	2.4
Nepal	18.0	9.1	26.9	3.0	-2.93	8.92	3.5	1.7	5.3
Niger	16.0	7.1	24.8	12.2	-0.12	24.48	4.0	1.7	6.2
Nigeria	64.3	60.0	68.6	26.8	17.82	35.83	23.2	21.0	25.3
Pakistan	31.4	22.8	40.1	2.6	-8.62	13.86	7.1	4.9	9.2
Papua New Guinea	28.7	18.3	39.1	9.3	-2.42	20.99	11.2	6.9	15.5
Peru	9.2	3.7	14.8	7.3	-1.36	16.02	1.8	0.7	2.9
Philippines	19.4	11.5	27.3	10.4	-4.04	24.90	4.1	2.4	5.8
Rwanda	1.0	-1.3	3.2	0.3	-2.64	3.17	0.2	-0.2	0.5
Senegal	9.4	0.5	18.3	0.2	-21.81	22.21	1.7	0.1	3.4
Sierra Leone	-1.6	-8.9	5.8	-3.2	-11.23	4.88	-0.3	-1.9	1.3
South Africa	4.4	-24.8	33.6	9.1	-21.11	39.27	1.3	-6.9	9.5
Tajikistan	2.2	-3.4	7.7	2.1	-7.08	11.37	0.4	-0.6	1.4
Timor-Leste	14.5	4.4	24.6	0.9	-14.87	16.59	4.0	1.2	6.8
Togo	12.7	2.0	23.3	6.1	-2.10	14.22	2.6	0.4	4.7
Uganda	5.9	-0.3	12.1	1.7	-4.92	8.41	1.3	-0.1	2.6
United Republic of Tanzania	13.8	5.8	21.8	0.7	-0.88	2.25	2.6	1.0	4.2
Zambia	9.5	3.4	15.6	6.2	-7.20	19.65	1.7	0.6	2.8
Zimbabwe	15.6	4.1	27.0	-0.5	-8.95	8.04	3.2	0.8	5.5

Table S5. Measures of inequality in zero-DTP prevalence by SWPER Global Index social independence domain, children age 12-23 months.

Country	Crude SII	LL	UL	Adjusted SII	LL	UL	CIX	LL	UL
Afghanistan	-10.3	-18.7	-2.0	-0.8	-3.2	1.6	-6.5	-11.9	-1.1
Angola	-28.0	-37.1	-19.0	-5.7	-20.6	9.2	-15.1	-20.1	-10.1
Armenia	-0.9	-4.1	2.4	-0.4	-1.4	0.7	-9.2	-42.1	23.8
Bangladesh	-1.8	-3.6	0.1	-1.6	-4.6	1.5	-20.2	-38.5	-2.0
Benin	-11.1	-17.7	-4.5	-1.5	-13.5	10.5	-11.3	-17.6	-5.0
Burkina Faso	-5.8	-9.4	-2.3	-1.4	-4.4	1.6	-17.5	-27.4	-7.6
Burundi	-1.2	-2.5	0.1	-1.0	-1.9	-0.1	-39.2	-71.6	-6.7
Cambodia	-9.7	-16.3	-3.0	-3.3	-9.4	2.9	-26.8	-45.1	-8.5
Cameroon	-19.3	-27.9	-10.7	0.2	-15.3	15.6	-19.1	-26.6	-11.5
Chad	-12.4	-20.4	-4.4	-8.4	-19.3	2.5	-4.9	-8.1	-1.7
Comoros	-12.3	-25.1	0.4	-5.7	-28.7	17.2	-12.1	-24.5	0.4
Côte d'Ivoire	-10.0	-22.1	2.1	-6.3	-26.4	13.8	-7.3	-15.9	1.3
Democratic Republic of the Congo	-17.5	-24.4	-10.6	-5.2	-12.4	2.1	-16.0	-22.4	-9.5
Dominican Republic	-12.8	-24.1	-1.6	-5.8	-11.9	0.3	-21.9	-42.2	-1.7
Egypt	-0.9	-2.4	0.6	-0.7	-2.0	0.6	-24.5	-61.2	12.2
Ethiopia	-24.8	-36.0	-13.6	-7.6	-20.5	5.3	-16.2	-23.8	-8.7
Gabon	-5.8	-18.5	6.9	8.5	-22.0	39.0	-10.1	-33.1	12.9
Gambia	1.5	-1.6	4.6	4.4	-15.3	24.2	14.9	-13.7	43.4
Ghana	-5.2	-9.5	-0.9	-3.8	-12.1	4.4	-27.4	-45.9	-8.9
Guatemala	-3.6	-7.1	-0.1	-5.5	-13.8	2.8	-23.6	-46.0	-1.2
Guinea	-5.4	-15.3	4.5	3.8	-8.9	16.5	-2.4	-6.8	2.0
Haiti	-18.4	-28.4	-8.4	-11.0	-29.6	7.7	-18.0	-26.9	-9.1
Honduras	1.0	-1.0	2.9	0.7	-1.9	3.4	18.7	-16.8	54.1
India	-10.1	-13.4	-6.9	-5.9	-13.3	1.6	-18.9	-24.9	-12.8
Indonesia	-10.9	-16.3	-5.5	-10.6	-23.4	2.1	-17.0	-24.5	-9.5
Kenya	-2.2	-4.6	0.3	0.1	-1.0	1.2	-20.7	-44.0	2.5
Kyrgyzstan	-2.1	-9.5	5.4	-0.9	-2.7	0.9	-20.9	-94.5	52.7
Lesotho	0.5	-3.5	4.4	1.8	-3.0	6.7	4.6	-33.2	42.3
Liberia	-10.8	-22.0	0.5	-2.5	-7.9	2.9	-18.8	-38.2	0.5
Malawi	-3.8	-6.5	-1.0	-2.3	-5.0	0.4	-27.2	-43.4	-10.9
Mali	-12.6	-19.8	-5.4	-2.7	-13.1	7.8	-11.7	-18.1	-5.3
Mozambique	0.3	-6.7	7.4	0.2	-3.8	4.1	0.6	-12.8	14.0
Myanmar	-20.8	-30.1	-11.6	-19.1	-45.5	7.4	-26.8	-36.0	-17.5

Namibia	9.3	-1.9	20.6	0.0	-0.3	0.2	26.1	-1.2	53.4
Nepal	-4.6	-9.1	-0.1	-0.3	-1.0	0.4	-22.8	-44.1	-1.5
Niger	-7.8	-14.2	-1.4	-5.7	-15.4	3.9	-9.5	-17.2	-1.7
Nigeria	-60.1	-64.4	-55.7	-28.4	-37.5	-19.3	-30.8	-33.0	-28.6
Pakistan	-23.9	-31.4	-16.5	-8.6	-28.3	11.0	-29.1	-36.6	-21.6
Papua New Guinea	-22.2	-34.2	-10.1	-13.6	-30.1	3.0	-10.9	-17.0	-4.8
Peru	-3.4	-7.1	0.4	-1.2	-4.0	1.6	-12.6	-26.5	1.3
Philippines	-14.2	-21.3	-7.1	-15.6	-34.1	2.9	-17.7	-26.2	-9.3
Rwanda	-0.5	-1.8	0.7	-1.0	-2.8	0.8	-20.3	-63.2	22.5
Senegal	-0.7	-6.9	5.6	7.6	-17.3	32.5	-2.7	-27.2	21.9
Sierra Leone	-3.1	-7.0	0.9	-3.4	-11.2	4.3	-10.1	-22.5	2.2
South Africa	-7.4	-25.3	10.5	1.3	-7.7	10.3	-13.5	-45.4	18.4
Tajikistan	-2.3	-5.5	0.9	-8.2	-25.9	9.5	-15.0	-33.6	3.6
Timor-Leste	-12.5	-20.4	-4.6	6.1	-7.5	19.7	-9.8	-15.9	-3.7
Togo	-8.8	-15.7	-1.8	-0.5	-1.8	0.7	-21.8	-39.9	-3.7
Uganda	-2.6	-6.2	1.0	-0.2	-2.3	2.0	-8.9	-21.0	3.2
United Republic of Tanzania	-6.8	-10.5	-3.1	-1.6	-3.9	0.6	-35.2	-50.9	-19.6
Zambia	-2.5	-5.4	0.4	-0.7	-3.4	1.9	-22.8	-48.2	2.6
Zimbabwe	-14.4	-24.6	-4.3	-0.5	-6.7	5.7	-22.9	-36.7	-9.1
